# DNA Self-Assembly of Targeted Near-Infrared-Responsive Gold Nanoparticles for Cancer Thermo-Chemotherapy**

**DOI:** 10.1002/anie.201204018

**Published:** 2012-10-18

**Authors:** Zeyu Xiao, Changwei Ji, Jinjun Shi, Eric M Pridgen, Jillian Frieder, Jun Wu, Omid C Farokhzad

**Affiliations:** Laboratory of Nanomedicine and Biomaterials, Department of Anesthesiology, Brigham and Women's Hospital, Harvard Medical SchoolBoston, MA, 02115 (USA); MIT-Harvard Center for Cancer Nanotechnology Excellence, Massachusetts Institute of TechnologyCambridge, MA, 02139 (USA); The David H. Koch Institute for Integrative Cancer Research and Department of Chemical Engineering, Massachusetts Institute of TechnologyCambridge, MA, 02139 (USA); Department of Urology, The Affiliated Drum Tower Hospital of Nanjing, University Medical SchoolNanjing, Jiangsu Province, 210008 (China)

**Keywords:** cancer, DNA, drug delivery, nanorods, near-infrared light

The development of external stimulus-responsive nanoparticle (NP) systems for cancer therapy has received considerable attention in recent years, as these systems can differentially increase drug accumulation at target cancer cells/tissues, drastically decrease systemic toxicity, and potentially avoid under- or over-dosing.^1^ External stimuli that have been exploited for such applications include light,^2^ magnetic field,^3^ ultrasound,^4^ and electricity.^5^ Among them, near-infrared (NIR) light (650–900 nm) has recently become an attractive stimulus because of its minimal absorbance by skin and tissue, thus allowing for noninvasive and deep tissue penetration.^6^ In particular, NIR light can be effectively converted into heat by using photothermal NPs, such as gold nanorods (NRs),^7^ gold nanoshells,^8^ hollow gold nanospheres,^9^ and carbon nanotubes.^10^ As such, NIR-responsive NP platforms offer several important benefits for cancer therapy. For example, NIR-induced local heating can be used for cancer thermotherapy.^11^ In addition, NIR-responsive NP delivery systems enable on-demand release of drugs for cancer chemotherapy, presumably by heat-induced disruption of the delivery vehicles.^7^, 9b Furthermore, the combination of NIR-based thermotherapy and triggered chemotherapy (thermo-chemotherapy) could provide higher therapeutic efficacy than respective monotherapies.9b, 12

In addition to these advantages, investigators are exploring the possibility of integrating active targeting ligands in NIR-responsive NP platforms for targeted cancer thermo-chemotherapy. This triple combination of thermotherapy, triggered drug release, and targeted delivery, would achieve optimal therapeutic efficacy in cancer treatment, relative to pairwise combinations. For example, Lee et al.^13^ have designed folate-conjugated, doxorubicin (Dox) loaded poly(lactic-co-glycolic acid) (PLGA)–gold half-shell NPs, and this combination led to effective tumor elimination in target tissues in a NIR-responsive manner. A current strategy in formulating this targeted NIR-responsive NP requires multiple steps, including 1) the synthesis of drug-loaded NPs, 2) deposition of gold compositions on NPs, and 3) post-conjugation with targeting ligands followed by purification. However, these complex processes could increase the difficulty of adjusting bio-physicochemical properties of NPs in a reproducible manner, and could contribute to unintended drug release from NPs, thereby resulting in unfavorable batch-to-batch variability in the characteristics of drug loading. Alternatively, using pre-functionalized components to self-assemble into targeted NPs would eliminate the need for post-modification of NPs and is amenable to being scaled-up with little batch-to-batch variability.^14^ This self-assembly strategy has led to the clinical translation of first-in-man targeted cyclodextrin-based NPs for small interfering RNA (siRNA) delivery,^15^ and targeted PLGA-based NPs for docetaxel delivery.^16^ Nevertheless, use of such a self-assembly strategy in the design of targeted NIR-responsive NPs has not been reported to date.

Inspired by nature and the ability of complimentary strands of DNA to hybridize, we designed a DNA-based platform that can self-assemble into targeted NIR-responsive NPs for cancer therapy. As illustrated in Figure 1, this platform comprises three distinct functional components: complementary DNA strands, the gold NR (50 nm×10 nm), and a polyethylene glycol (PEG) layer. The DNA strands, which consist of sequential CG base pairs, provide loading sites for Dox,^17^ a model chemotherapeutic drug. By changing the number of CG base pairs, drug loading can be precisely tuned. In addition to serving as drug-loading scaffold, one strand of the DNA (termed capture strand) is thiolated for gold NR capture, and the complementary strand (termed targeting strand) is pre-conjugated with ligands for cell-specific targeting. Gold NRs serve as the model NIR light-to-heat transducer for cancer thermotherapy^18^ and for denaturing the DNA double helix upon NIR irradiation,^19^ leading to the triggered release of loaded drugs at target site for chemotherapy. The PEG layer allows the NPs to evade recognition by the immune system and prolongs the circulation half-life of the NPs.^15^ Most notably, the assembly of this multifunctional platform can be achieved by DNA hybridization in a single step, which contributes to the tunable and predictable feature in targeting and drug loading. Besides, these DNA-assembled NPs have a relatively small size (about 10–100 nm), which could facilitate their extravasation out of circulation at the tumor site^20^ and diffusion in the tumor extracellular space,^21^ thus enhancing the anti-tumor efficacy.

**Figure 1:**
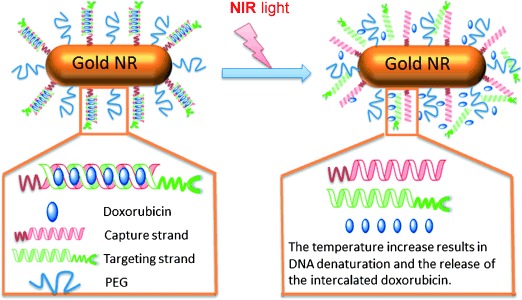
DNA assembly of a targeted, NIR-responsive delivery platform. This platform comprises gold NRs (50 nm×10 nm), PEG layers, and complementary DNA oligonucleotides consisting of capture strands and targeting strands. Consecutive CG base pairs provide binding sites for doxorubicin (Dox) loading. The capture strands are conjugated to gold NRs for NIR response. The targeting strands are complementary to the capture strands and conjugated with ligands for molecular targeting. The delivery platform is assembled through the hybridization of capture strands attached on the NRs and targeting stands. The resulting double-stranded DNA structures form scaffolds for Dox intercalation. Upon NIR irradiation, the heated gold NRs result in DNA denaturation and the release of drugs (Dox) at the target site.

We first designed the DNA sequence with 24-base pair (CGA)_8_/(TCG)_8_ for Dox loading, as this anti-cancer drug can preferentially intercalate into double-stranded CG base pair.^17^ Previous studies have shown that the fluorescence of Dox can be quenched after intercalation into the CG base pair;^22^ we used this finding to monitor the number of Dox molecules loaded onto the designed 24-base-pair DNA strands. Figure 2 shows a sequential decrease in the Dox fluorescence intensity, when a fixed concentration of Dox was incubated with an increasing molar ratio of the double-stranded (CGA)_8_/(TCG)_8_. Ultimately, a maximum level of fluorescence quenching was reached, indicating that the loading capacity of the designed DNA sequence was 7.5 Dox per (CGA)_8_/(TCG)_8_ duplex.

**Figure 2:**
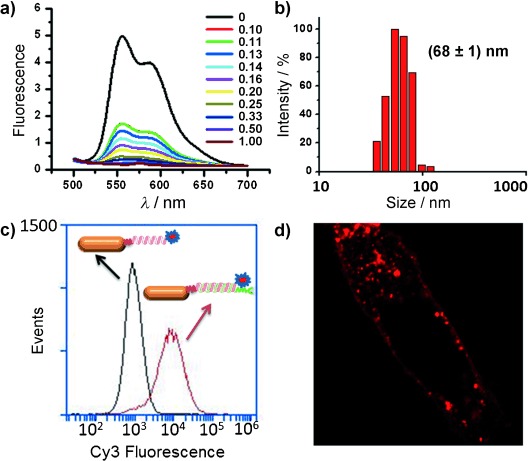
a) Fluorescence spectra of the Dox (1 μM) solution with increasing molar ratio of hybridized DNA duplex (from top to bottom: 0, 0.10, 0.11, 0.13, 0.14, 0.16, 0.20, 0.25, 0.33, 0.50, and 1.00 equiv) at an excitation of 480 nm. b) Hydrodynamic size distribution of T-DNA-NR showing the mean value at 68 nm. c) Flow cytometry profile of cellular binding and uptake of nontargeted ONT-NR-Cy3 (black line) and targeted T-DNA-NR-Cy3 (red line) in KB cells. d) A representative confocal image showing the cellular distribution of T-DNA-NR-Cy3.

We next synthesized and characterized the targeting strands and the gold NR-conjugated capture strands. The targeting strand was constructed by conjugating *N*-hydroxysuccinimide (NHS) terminated (TCG)_8_ oligonucleotide (ONT) with NH_2_-terminated PEG-folic acid (FA).^23^ The anion exchange chromatography analysis (see Figure S1 in the Supporting Information) confirmed the successful conjugation of (TCG)_8_-PEG-FA. Free (TCG)_8_ ONT was eluted at a retention time of 10.7 min, whereas the (TCG)_8_-PEG-FA conjugate was eluted with two peaks at 8.7 and 8.9 min, which represent the β and γ carboxyl groups on FA, respectively.^23^ The capture strand, which is complimentary to the targeting strand, was constructed by modifying (CGA)_8_ ONT with ethylene glycol-thiol at its 5′ end, wherein the thiol group can capture the gold NR surface by the thiol–gold bond,^24^ and the ethylene glycol segment can separate the alkanethiol from the capture strand, and thus avoid possible steric hindrance during the hybridization of capture/targeting strand.^25^ Notably, to minimize the aggregation and nonspecific protein binding in vivo,^26^ the gold NR surface was pre-assembled with a thiolated PEG layer, and then mixed with capture strands to form gold NR-capture strand conjugates. To quantify the surface coverage of the capture strands on gold NRs, Cy3-labeled capture strands with different concentrations were reacted with NRs, and a rinse cycle was followed to remove the nonchemisorbed strands. The Cy3-labeled strands chemisorbed on the NRs were then displaced by mercaptoethanol,^27^ and were quantified using fluorescence spectroscopy by interpolation from a standard liner calibration curve. Totally, about 72 capture strands were bound on each gold NR (Figure S2).

NR-conjugated capture strands (ONT-NR) were mixed with targeting strands, and DNA hybridization led to the assembly of the targeted DNA gold NR (T-DNA-NR) platform. Dynamic light scattering showed the T-DNA-NR had a hydrodynamic size of 68±1 nm (Figure 2 b), compared to gold NR which had a hydrodynamic size of 46±2 nm (data not shown). Additionally, the T-DNA-NRs remain stable in the cell-growth medium without a significant change in size over a period of two days, indicating their potential for in vivo applications.

To demonstrate the capacity of T-DNA-NR to interact with target cells, we visualized the cellular binding and uptake of Cy3-labeled T-DNA-NRs (T-DNA-NR-Cy3). Human nasopharyngeal epidermoid carcinoma (KB) cells, which overexpress folate receptors, were incubated at 37 °C for 2 h with nontargeted ONT-NR-Cy3 and FA-targeted T-DNA-NR-Cy3, and subsequently washed to remove unbound bioconjugates. Compared with ONT-NR-Cy3, T-DNA-NR-Cy3 showed a 10-fold increase in its binding and uptake fluorescence profile, as evidenced by flow cytometric analysis (Figure 2 c). High-magnification confocal microscopy further confirmed the effective uptake of the T-DNA-NR-Cy3 (Figure 2 d). These results demonstrate the potenital of our T-DNA-NRs for efficient targeted drug delivery.

Next, we loaded Dox onto the T-DNA-NR platform (T-DNA(Dox)-NR) through intercalation with GC base pairs, and examined its release upon NIR irradiation in vitro. Based on the loading of 7.5 Dox molecules per hybridized (TCG)_8_/(CGA)_8_ pair, and 72 copies of ONTs per NR, we inferred that each T-DNA-NR can load about 576 Dox molecules. The capablity of gold NRs to generate heat upon NIR irradiation was examined by inserting the probe of a thermometer into the medium with NRs. As shown in Figure S3, the temperature of the medium increased and reached about 80 °C at 2.5 min of irradiation (laser power: 600 mW), indicating the efficacy of the NRs to elevate the temperature of surrounding envioronments. To study the Dox release, KB cells were incubated with T-DNA (Dox)-NR for 2 h, and excess nanoconjugates were removed; T-DNA (Dox)-NRs bound to or taken up into the target cells were then exposed to NIR irradiation for 0, 5, and 10 min. The fluorescence of Dox molecules was initially quenched because of their intercalation into DNA helices. Upon NIR illumination, the photothermal heating on the NR surface caused the denaturation of DNA helices at their melting temperature,^19^ leading to the release of Dox molecules into the cells. The number of released Dox molecules, reflected by the fluorescence of the whole cell, was assessed by flow cytometry analysis (Figure 3). Notably, a right shift of the fluorescence profile was also observed in the sample that was not NIR irradiated (NIR 0 min), attributable to unquenched background Dox fluorescence and a few Dox molecules liberated from the conjugates by diffusion during the 2 h of incubation.^22^ Following 5 min of irradiation, the fluorescence signal of the Dox molecules was observably increased, indicating the partial release of the drug. After 10 min of irradiation, most Dox molecules were liberated from the DNA double helices, resulting in a distinct right shift of the fluorescence profile.

**Figure 3:**
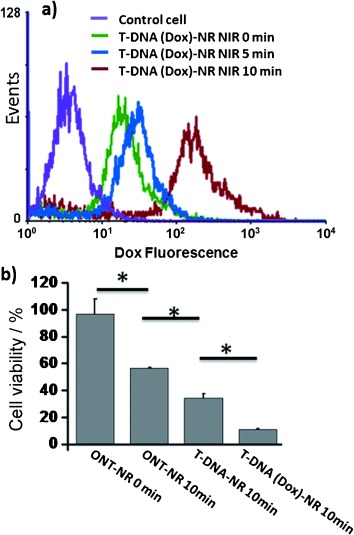
a) NIR-responsive Dox release from T-DNA(Dox)-NR in vitro. Flow cytometry histogram profile of Dox fluorescence in KB cells upon different NIR irradiation time. b) In vitro cell viability measured by MTT assays. KB cells were incubated with ONT-NR, T-DNA-NR, and T-DNA(Dox)-NR for 2 h. Cells were then washed, exposed to NIR for 10 min, and incubated additionally 48 h prior to the cell viability measurement. *, *P*<0.05 by a two-sample student’s t-test.

We used 10 min as the optimized irradiation time, and compared the therapeutic effects of different NP formulations in vitro. KB cells were incubated with the same dose of ONT-NR, T-DNA-NR, and T-DNA(Dox)-NR for 2 h, washed twice, irradiated by NIR for 10 min, and further incubated with fresh media for 48 h. Cell viability was evaluated by the 3-4,5-dimethylthiazol-2-yl-2,5-diphenyl tetrazolium bromide (MTT) assay. As demonstrated in Figure 3 b, T-DNA-NR showed greater cytotoxicity than ONT-NR (34.37±3.03 versus 56.37±0.69, mean value±standard deviation, (mean±SD), *n*=3, *P*<0.05), because of a higher amount of FA-targeted T-DNA-NR bound to and internalized by KB cells, compared to the amount of nontargeted ONT-NR. T-DNA(Dox)-NR, which releases Dox molecules during NIR irradiation, provided the highest cytotoxicity (10.77±0.57, mean±SD, *n*=3, *P*<0.05).

We proceeded to explore the capability of T-DNA(Dox)-NR to respond to NIR stimuli and release of Dox molecules in vivo. Previous studies suggest that NP attachment and Dox intercalation markedly improves the stability and resistance of nucleotides to enzyme degradation in vivo,^28^ which allowed us to apply the T-DNA(Dox)-NR platform in vivo without the requirement for degradation-resistant DNA modification. Xenograft tumor models were developed by injecting KB cells subcutaneous (s.c.) in the flank of BALB/c nude mice. After the tumor size reached about 100 mm^3^, a single intratumoral injection of T-DNA(Dox)-NR was administered, and 2 h later, the mice were divided into four groups, and the tumor region was exposed to NIR laser light for 0, 5, 10, or 15 min (4 mice per group). Each tumor was then collected, frozen, and four 10 μm thick slices were taken from the mid-cross section of the tumor tissue. Thus, a total of 16 slices were processed for statistical analysis in each group. Because released Dox molecules can diffuse into the tumor cells and yield red fluorescence, the average fluorescent intensity of each tumor slice, as measured on confocal microscopy, was used as an indication of the amount of released Dox molecules. Compared to the 0 min-irradiation group, which shows the average fluorescence intensity at 19.45±4.79 (mean value±standard error, (mean±SE), *n*=16), the 5 min-irradiation group showed a three-fold increase in intensity (86.78±25.46; mean±SE, *n*=16, *P*<0.05); and the 10 min- and 15 min-irradiation groups similarly showed a 39-fold increase in intensity (757.30±124.18 and 753.97±123.02, respectively; mean±SE, *n*=16, *P*<0.001; Figure S4). Representative histological sections were photographed to document the differential Dox release upon different irradiation times (Figure S4a), with a maximal release at 10 min.

Finally, to demonstrate the robust and reproducible features of our NP platform, we evaluated its in vivo anti-tumor efficacy in two folic receptor-overexpressed tumor models (KB and HeLa). Subcutaneous tumors were initiated in the flank of the BALB/c nude mice by injecting one million KB or HeLa-Luciferase (HeLa-Luc) cells. After the tumors had developed to about 100 mm^3^, the drug efficacy was studied in four groups of mice (*n*=7 per group), with weight and tumor size differences minimized among the groups. Four regimens (phosphate buffered saline (PBS), ONT-NR, T-DNA-NR, and T-DNA(Dox)-NR) were administered by a single intratumoral injection, with a low dose (1.5×10^10^ NR particles per mouse) in the KB tumor model and a high dose (4.5×10^10^ NR particles per mouse) in the HeLa-Luc tumor model. Two hours post injection, the tumor of each mouse was irradiated for 10 min (600 mW, 808 nm), and tumor development was monitored by measuring the tumor size (KB tumor model) or by measuring bioluminescent imaging (HeLa-Luc tumor model) at regular intervals for two weeks. In the KB tumor model, the mean tumor volumes at different days were calibrated by normalizing the initial volume (at day 0) to 1 (Figure 4). At the end of the experiment (day 14), the relative tumor volume of the ONT-NR group was 3.93±0.40, that is, 24 % less than that of the PBS group (5.21±0.42). The relative tumor volume of T-DNA-NR group was 2.52±0.58, 35 % less than that of the ONT-NR group; and the relative tumor volume of the T-DNA (Dox)-NR group was 1.82±0.25, 28 % less than that of the T-DNA-NR group (mean±SE, *n*=7, *P*<0.05). For HeLa-Luc tumor model, the total bioluminescence intensity (photons/sec) obtained from each tumor at different days was calibrated by normalizing initial bioluminescence signal (at day 0) to 1. The relative luminescence intensity signal (mean±SE, *n*=7) was then plotted as a function of time (Figure 5), to indicate the time course of the tumor growth. On day 12, the ONT-NR group showed a relative signal of 6.04±0.88, 41 % less than PBS group (10.18±2.88); the T-DNA-NR group showed a relative signal of 1.98±0.49, 64 % less than the ONT-NR group; and the T-DNA(Dox)-NR group showed a relative signal of 0.58±0.26, 71 % less than the T-DNA-NR group. Consistent with previous observations,^13^, ^29^ the treatment with ONT-NR delayed tumor growth compared to treatments with PBS, due to the photothermal ablation of cancer cells. Moreover, the T-DNA-NR group showed significantly higher efficacy in tumor reduction compared to ONT-NR group, presumalby because the FA targeting property allows a higher amount of T-DNA-NRs to bind to the cell surface and to be internalized by the KB/HeLa-Luc cells, whereas the non-targeted ONT-NRs only diffuse into the extracellular space between tumor cells, and are endocytosed to a very limited extent. In addition, because heat transfer from the surface of NRs to the surrounding cellular environment is highly localized and decays exponentially within a few nanometers,^19^ targeted T-DNA-NRs, which had a shorter distance to the cancer cell surface than non-targeted ONT-NRs, led to an enhanced destruction of cancer cells. Furthermore, Dox molecules that are released from T-DNA(Dox)-NRs subsequent to NIR irridiation contributed to the additional chemotherapeutic efficacy of T-DNA(Dox)-NRs in tumor reduction relative to T-DNA-NR treatment alone. Notably, the T-DNA(Dox)-NR group without NIR irradiation also demonstrated modest tumor reduction compared to the PBS group, presumbly because of the gradual release of dox molecules from the intratumorally injected T-DNA-NR complex overtime.

**Figure 4:**
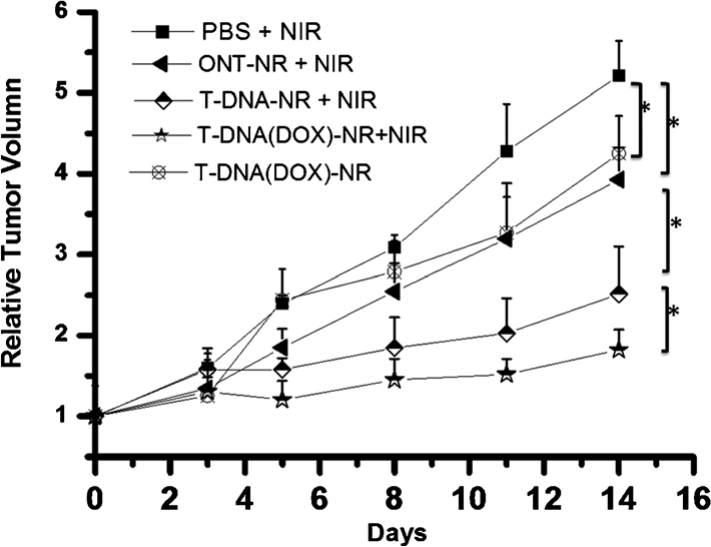
Anti-tumor effects of various treatments on KB tumor-bearing mice. ONT-NR, T-DNA-NR, and T-DNA(Dox)-NR were injected intratumorally in a single dose (1.5×10^10^ NR particles), followed by 10 min NIR irradiation or without NIR irradiation. The volumetric changes in tumor size relative to that at day 0 are plotted over time after irradiation. Data are presented as mean±SE of seven mice per group. *, *P*<0.05 by two-sample student’s t-test.

**Figure 5:**
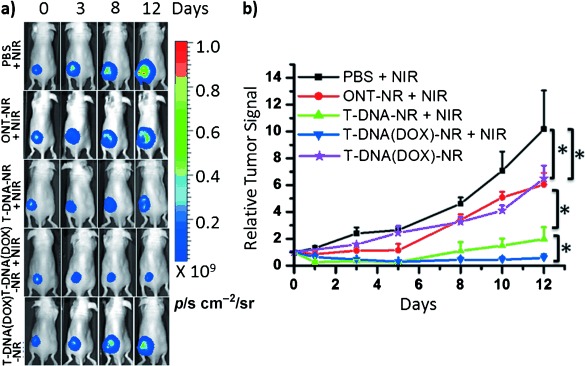
Anti-tumor efficacy of the NIR-responsive NP platform on HeLa-Luc tumor-bearing mice. ONT-NR, T-DNA-NR, and T-DNA(Dox)-NR were injected intratumorally in a single dose (4.5×10^10^ NR particles), followed by 10 min of NIR irradiation or without NIR irradiation. Images were taken at day 0, 1, 3, 5, 8, 10, and 12, respectively. The changes in luminescence intensity indicate the tumor growth. a) Representative mice images showing the tumor progression under different treatment conidtions. b) The tumor luciferase intensities relative to day 0 are plotted over time after NIR irradiation. Data are presented as mean±SE of seven mice per group. *,*P*<0.05 by two-sample student’s t-test.

In summary, we have developed a targeted NIR-responsive NP delivery platform by a simple DNA self-assembly process. The in vitro and in vivo results demonstrate that this platform selectively delivers anti-cancer drugs to target cells, releases them upon NIR irradiation, and effectively inhibits tumor growth through thermo-chemotherapy. Despite the use of intratumoral injection of NPs in the current study, this local delivery strategy serves as an initial step to test the efficacy of our DNA-assembled NP platform, and further systematic delivery of NPs will be explored to expand our findings. In particular, our platform incorporates the targeting ligands through a DNA-assembly process and loads drugs thereafter, and thus the ligand density and drug loading can be fine-tuned and precisely controlled, which will facilitate the optimization of NP bio-physicochemical properties to achieve optimal biodistribution for systemic adminstration. We also anticipate that the present system could be accommodated with different therapeutics, and could be similarly incorporated with other NIR transducers and disease-specific targeting ligands for the treatments of a myriad of important human diseases.

## Notes

Detailed facts of importance to specialist readers are published as ”Supporting Information”. Such documents are peer-reviewed, but not copy-edited or typeset. They are made available as submitted by the authors.
