# Supplementary material for: DNA Self-Assembly of Targeted Near-Infrared-Responsive Gold Nanoparticles for Cancer Thermo-Chemotherapy
Source: Angew Chem Int Ed Engl. 2012 Oct 18;51(47):11853–7. doi: 10.1002/anie.201204018 (PMC3532659; doi:10.1002/anie.201204018)
Supplement: Supplementary file 1 [file anie0051-11853-sd1.pdf]

Angewandte Chemie
Eine Zeitschrift der Gesellschaft Deutscher Chemiker

Supporting Information

© Wiley-VCH 2012

69451 Weinheim, Germany

DNA Self-Assembly of Targeted Near-Infrared-Responsive Gold Nanoparticles for Cancer Thermo-Chemotherapy**

*Zeyu Xiao, Changwei Ji, Jinjun Shi, Eric M. Pridgen, Jillian Frieder, Jun Wu, and Omid C. Farokhzad**

anie_201204018_sm_miscellaneous_information.pdf

Supporting Information

Part I: Experimental Section

Materials and Cell lines. Cetyltrimetylammonium bromide (CTAB), and tris(2-carboxyethyl) phosphine (TCEP) were obtained from Sigma-Aldrich (St. Louis, MO). Thiol-terminated methoxypoly(ethylene glycol) (mPEG-SH, Mw 5000) was purchased from Nanocs (New York, NY).

KB cells were maintained in RPMI1640 medium (Invitrogen) supplemented with 10 v/v% fetal bovine serum (FBS, Sigma) and 1 v/v% penicillin/streptomycin (Sigma). A HeLa cell line stably expressing firefly and Renilla luciferase (HeLa-Luc) was obtained from Alnylam Pharmaceuticals, Inc., and were maintained in Dulbecco's Modified Eagle's Medium (DMEM, Invitrogen) with high glucose, 10 v/v% FBS, 500 μg/mL zeocin, and 0.5 μg/mL puromycin.

Measurement. The hydrodynamic particle size of T-DNA-NR was measured using a ZetaPALS dynamic light-scattering detector (15 mW laser, incident beam = 676 nm; Brookhaven Instruments). Dox fluorescence was monitored at an excitation wavelength of 480 nm, and emission was recorded over a 500-700 nm interval (3 mm slit) on an RF-5301PC spectrofluorometer.

Functionalization and characterization of capture ONT-NRs. Highly stable solutions of CTAB-coated gold NR (~ 50 nm × 10 nm in size) with longitudinal plasmon resonance at 808 nm (Nanopartz, a division of Concurrent Analyticl, Inc.) were subjected to two wash–centrifugation cycles at 12000 rpm for 5 min to remove excess CTAB. Before conjugation with gold NRs, the thiol functionality on the capture ONT (5'-/ThioMC6-D/CGA CGA CGA CGA CGA CGA CGA CGA-3') was deprotected by 5 mM of TCEP (Neutral pH, Thermo Scientific) in PBS (PH=7.4) for 30 min at room temperature. To stabilize and functionalize the gold NRs, fresh mPEG-SH stock solution (2.0 mM) was added to the NRs suspension (1 nM, 1.0 mL). The mixture sat for 1 h at room temperature, followed by addition of alkanethiol capture ONT (100 nM). Mixtures were incubated for 12 h, after which the solution was buffered at pH 7 (10 mM phosphate), and NaCl solution was added (final concentration of 0.1 M); the entire mixture was allowed to "age" under these conditions for additional 12 h, and excess reagents were finally removed by centrifugation at 12000 rpm for 5 min.

The loading of capture ONT on the NR surface was measured on fluorescence spectrophotometer (HITACHI F-2700). Capture ONTs were labeled with Cy3 at the 3' end (5'-/ThioMC6-D/CGA CGA CGA CGA CGA CGA CGA CGA /Cy3/-3', Cy3-ONTs) and were similarly functionalized with NRs with different ONT-NR ratios. After extensive washing to remove the excess non-conjugated ONTs, mercaptoethanol (12 mM) in 0.3 M PBS was added to Cy3-ONT-modified NRs. The mixture was incubated for 18 h at room temperature with intermittent shaking, and the solutions containing displaced Cy3-ONTs were separated by centrifugating the NR aggregates. The fluorescence maximums

(measured at 580 nm) of the supernatant, containing displaced Cy3-ONTs from NR surfaces, were converted to molar concentrations of the Cy3−ONTs by interpolation from a standard linear calibration curve. Standard curves were prepared with known concentrations of Cy3-ONTs using identical buffer pH, salt, and mercaptoethanol concentrations. Finally, the average number of ONTs per particle was obtained by dividing the measured ONT molar concentration by the original NR concentration.

Synthesis of targeting strand ONT-PEG-FA. The NH_2-PEG-FA was firstly synthesized and analyzed as previously described.[1] CPG beads bound to NHS-Carboxyl-dT modified targeting ONT (5'-/NT/CG TCG TCG TCG TCG TCG TCG TCG -3', 80 mg) was mixed for 24 h with NH_2-PEG-FA (6 mg) dissolved in DMSO (200 uL) and triethylamine (20 uL) with gentle stirring. The mixture was centrifuged and washed three times with DMSO to remove the excess NH_2-PEG-FA in the supernatant. The DMT protecting group was removed from CPG beads by treating 80% acetic acid for 2 min at room temperature. The CPG beads were washed twice with acetonitrile, followed by volatilization. The targeting ONT-FA conjugates were cleaved from CPG beads and deprotected in 40% methylamine at 55 ℃ for 1 h, followed by ethanol precipitation. The products were analyzed and purified with anion exchange chromatography (Oligofactory Inc.).

Binding and uptake analysis of assembled T-DNA-NRs. Capture ONTs labeled with Cy3 at its 3' end (5'-/ThioMC6-D/CGA CGA CGA CGA CGA CGA CGA CGA /Cy3/-3', Cy3-ONTs) were conjugated with NRs as described above to yield ONT-NR-Cy3. Targeting strands were further incubated with ONT-NR-Cy3 to yield T-DNA-NR-Cy3. KB cells (10^5) were incubated with ONT-NR-Cy3 and T-DNA-NR-Cy3 for 2 h, followed by extensive washing to remove the unbound NRs. For flow cytometry analysis, cells were then detached by non-enzymatic cell dissociation solution (Invitrogen Corporation, Carlsbad, CA). The cell suspension was transferred into FACS tube with 40-μm cell strainer cap (Becton, Dickinson and Company, Franklin Lakes, NJ), and placed on ice before cytometric measurement. The fluorescence was determined with a FACScan cytometer (Accuri C6 Cytometers, Ann Arbor, MI) by counting 10000 events. For confocal imaging, cells were fixed with 4% formaldehyde, and cells were imaged on an Olympus FV500-IX81 confocal microscope (Olympus America Inc., Melville, NY) equipped with a 60× oil immersion objective (NA=1.40, Olympus, Melville, NY). A 488-nm He-Ne laser was used for excitation, and BP580±20 nm/BP520±12 fitler for emission.

NIR-responsive drug release *in vitro and in vivo*. KB cells (3×10^5/well, 24 well plate) were incubated with Dox loaded T-DNA(Dox)-NRs (2.2×10^5 NR particles) for 2 h, followed by washing twice. The cells were then irradiated with a CWdiode laser (Thorlabs, Newton, NJ) centered at 808 nm with an output power of 600 mW for 5 min or 10 min, separately. The cells were then detached by nonenzymatic cell dissociation solution, transferred into FACS tube with 40-μm cell strainer cap, and placed on ice for flow cytometric analysis. For *in vivo* experiments, xenograph flank tumors were induced in 8-week-old BALB/c nude mice by s.c. injection of one million KB cells suspended in 1:1

media and matrigel. Two weeks later, when tumors had reached ~100 mm^3, the mice were anesthetized with isofluorane, and tumors were intratumorally injected T-DNA(Dox)-NR (1.5×10^{10} NR particles). After 2 h, the flank tumor of each mouse was exposed to irradiation (~600 mW, 808 nm) for 0 min, 5 min, 10 min, or 15 min (n=4 mice per group). The tumors were then collected, snap frozen and immediately cryosectioned on a cryostat at a 10-μm thickness. Tissue slices were examined under an Olympus FV500-IX81 confocal microscope (Olympus America Inc., Melville, NY). To quantify Dox fluorescence, tumor slices were placed under a 10× oil immersion objective. The average fluorescence intensity of each slice image was analyzed using the region-of-interest function of the FluoView software. The 60× oil immersion objective was used for high magnification images.

***In vitro* cytotoxicity assays.** Cells (3×10^5) were seeded in 48-well plates, allowed to attach for 24 h, and exposed to ONT-NR, T-DNA-NR, T-DNA(Dox)-NR (2.2×10^5 NR particles) for 2 h. After washing, cells were then irradiated with NIR light (600 mW, 808 nm) for 10 min. Control cells were incubated in ONT-NR without NIR irradiation. All cells were incubated for an additional 48 h, after which MTT assays were used to determine cell viability. Data are presented as the mean ± SD of triplicate measurements.

***In vivo* anti-tumor efficacy studies.** All procedures with animals were carried out by a certified contract research organization; protocols used with live animals were consistent with local, state, and federal regulations as applicable, and were approved by the Institutional Animal Care and Use Committee. Xenograph flank tumors were induced in 8-week-old BALB/c nude mice (Charles River Laboratories International, Inc. Wilmington, MA) by s.c. injection of one million KB or HeLa-Luc cells, suspended in a mixture of 1:1 media and matrigel. After 2 weeks, when tumors had reached ~100 mm^3, mice were divided into four groups (n=7 per group; minimizing differences in weight and tumor size. Mice were anesthetized with isofluorane, intratumorally injected within the tumor of PBS, ONT-NR, T-DNA-NR, and T-DNA(Dox)-NR; 1.5×10^{10} NR particles were used for KB tumor model and 4.5×10^{10} NR particles were used for HeLa-Luc tumor model. Two hours post-injection, tumors were irradiated with or without NIR light (600 mW, 808 nm) for 10 min. For the KB tumor model, tumor size was monitored every 2 days for 2 weeks; length and width of the tumors were measured with use of digital calipers, and tumor volume was caculated according to the following formula: ($width^2 \times length / 2$). For the HeLa-Luc tumor model, the mice were monitored by a cryogenically cooled IVIS 100 Imaging System (Xenogen Corporation, 8 Alameda, CA) equipped with LivingImage acquisition and analysis software. Tumor bioluminescence was imaged every 2 days for two weeks. The average intensity of tumor luminescence signal was analyzed with use of the region-of-interest function of the software.

Part II: Figures

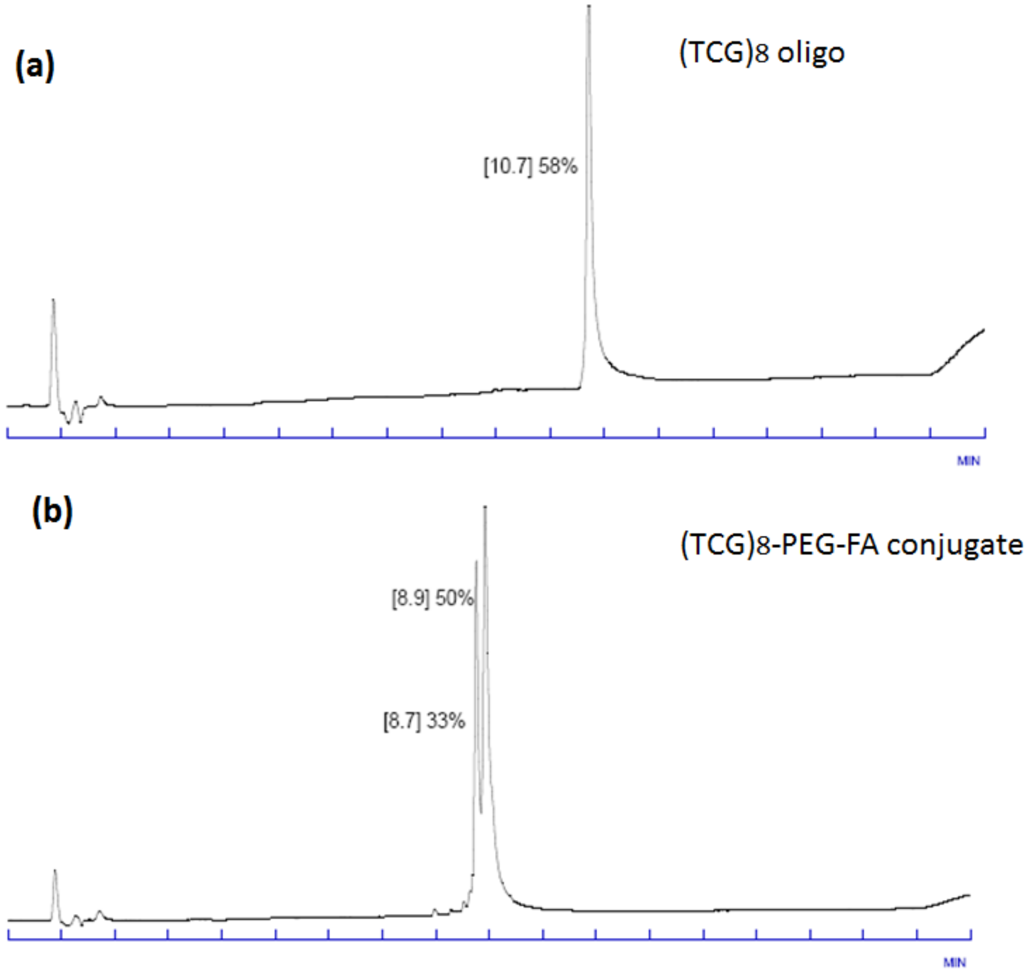

Figure S1. Anion exchange chromatography analysis of the conjugation of $(TCG)_8$-PEG-FA. (a) Free $(TCG)_8$ elutes at 10.7 min. (b) The conjugate $(TCG)_8$-PEG-FA elutes at 8.7 and 8.9 min, corresponding to the two conjugation isomers in PEGlated FA.

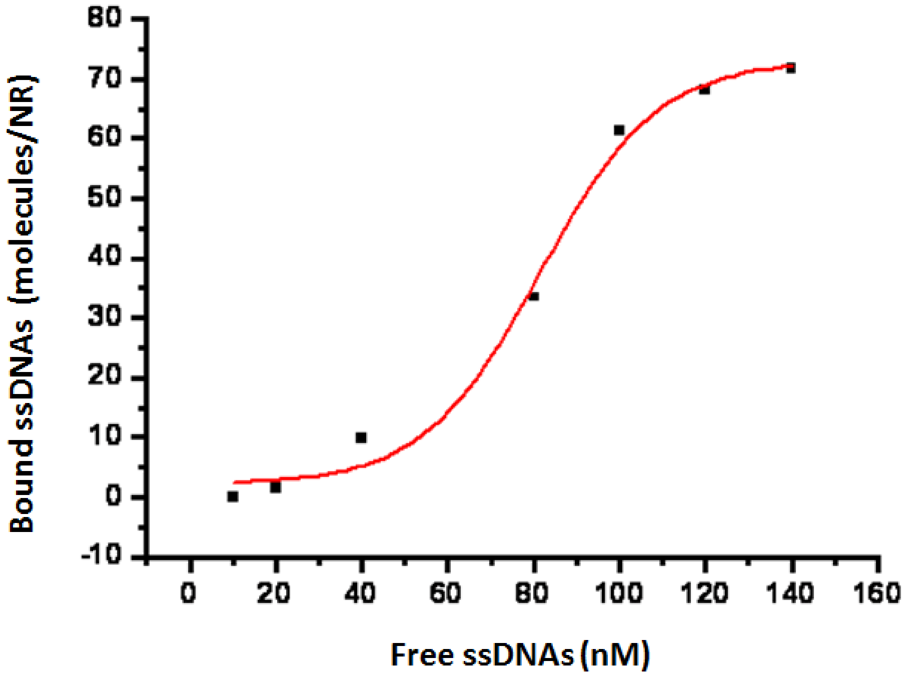

Figure S2. Saturation curve obtained by exposing gold NRs (1nM) to 0-160 nM cy3-labeled ssDNAs. Approximately 72 ssDNA molecules were bound on each gold NR.

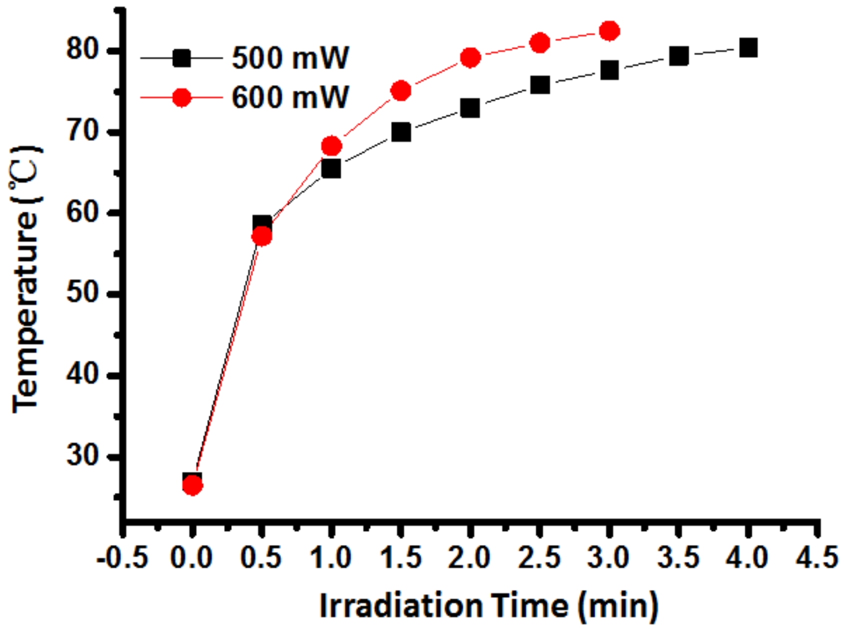

Figure S3. The temperature curve of gold NRs-contained medium upon different time period of NIR irradiation with the laser power of 500 mW (black curve) and 600 mW (red curve).

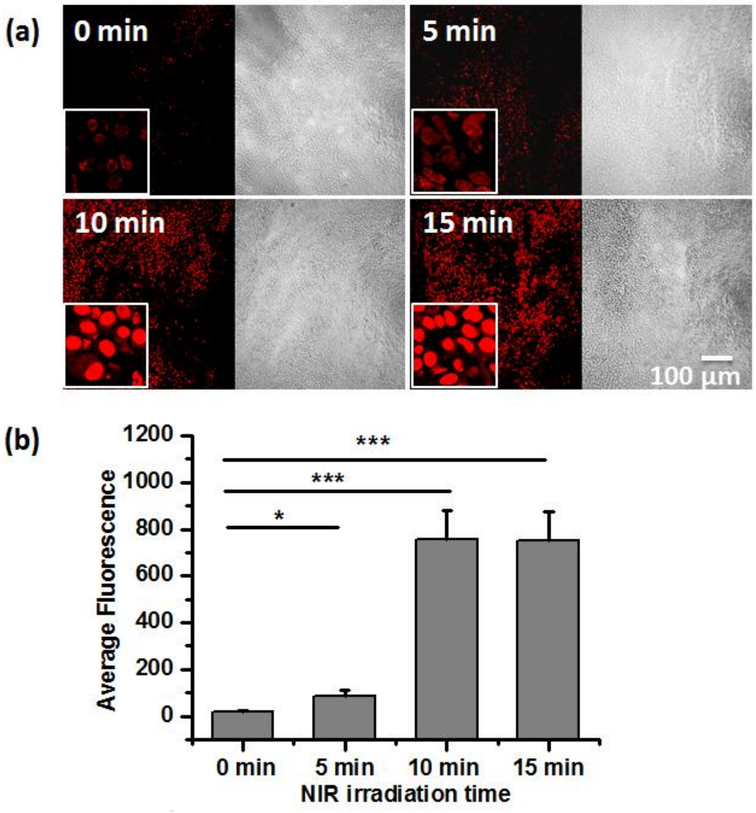

Figure S4. NIR-responsive Dox release from T-DNA(Dox)-NR in vivo. (a) Representative images of Dox release from T-DNA(Dox)-NR conjugates in tumor tissues upon NIR irradiation for 0 min (upper left), 5 min (upper right), 10 min (lower left), and 15 min (lower right). Red fluorescence indicates the released Dox molecules. In each image, left is the fluorescence image, right is the optical image, and small window is the magnified fluorescence image. (b) Statistics analysis of tissue fluorescence due to Dox release after differential NIR irradiation periods (*, $P<0.05$; ***, $P<0.001$ by student's t-test, Mean ± SE, n=16 per group).

[1] W. Gao, R. Langer, O. C. Farokhzad, *Angew Chem Int Ed Engl* **2010**, *49*, 6567-6571.
